# Anesthetic Management of Pregnant Patients With Cardiovascular Disease: A Practical, Lesion-Based Clinical Review

**DOI:** 10.7759/cureus.117526

**Published:** 2026-10-06

**Authors:** Alexander Iosovich, Alexander Ronenson, Emilia Guasch, Nicolas Brogly, Yahya Aljuba, Daniel Shatalin

**Affiliations:** 1 Department of Anesthesiology, Perioperative Medicine, and Pain Treatment, Shaare Zedek Medical Center, The Hebrew University of Jerusalem, Jerusalem, ISR; 2 Department of Anesthesia and Intensive Care, Hospital Universitario Fundación Jiménez Díaz, Madrid, ESP; 3 Department of Anesthesiology and Resuscitation, Gregorio Marañón General University Hospital, Madrid, ESP; 4 Department of Anesthesiology, Sanitas La Zarzuela University Hospital, Madrid, ESP; 5 Faculty of Medicine, Universidad Centro de Estudios Universitarios (CEU) San Pablo, Madrid, ESP

**Keywords:** cardiac disease, general anesthesia, hemodynamic management, maternal cardiac disease, multidisciplinary care, neuraxial anesthesia, obstetric anesthesia, postpartum care, pregnancy, risk stratification

## Abstract

Cardiovascular disease (CVD) is a major cause of maternal morbidity and mortality and increasingly complicates pregnancy as more women with congenital and acquired heart disease reach reproductive age. Anesthetic management of these patients requires integrating lesion-specific cardiovascular physiology, pregnancy-related hemodynamic changes, obstetric considerations, and the anticipated effects of labor, anesthesia, delivery, hemorrhage, and the postpartum transition. Despite contemporary cardiovascular and obstetric guidelines, translating lesion-specific physiology into practical anesthetic decisions remains challenging.

This narrative review provides a practical framework for the peripartum anesthetic management of pregnant patients with CVD. We discuss contemporary risk assessment using the modified World Health Organization 2.0 classification and the Cardiac Disease in Pregnancy II risk model, followed by principles of Pregnancy Heart Team planning, labor analgesia, cesarean delivery, monitoring, anticoagulation, uterotonic therapy, and postpartum surveillance. Lesion-specific considerations are reviewed for mitral stenosis, aortic stenosis, aortic disease and coarctation, pulmonary hypertension, pregnancy-associated myocardial infarction, peripartum cardiomyopathy, congenital heart disease, and Fontan circulation. Particular attention is given to maintaining appropriate preload, ventricular filling, systemic vascular resistance, cardiac rhythm, and oxygenation while avoiding abrupt changes in vascular resistance or cardiac loading conditions.

The review emphasizes individualized anesthetic planning rather than universal preferences for a particular anesthetic technique or mode of delivery. Neuraxial anesthesia can be advantageous in selected patients but should be considered in the context of ventricular function, pulmonary pressures, anticoagulation, urgency, and the expected hemodynamic response. General anesthesia remains an important option when neuraxial anesthesia is contraindicated or inadequate, rapid delivery is required, airway or ventilatory control is necessary, or maternal or surgical circumstances dictate. Uterotonic therapy should similarly be individualized according to cardiovascular physiology, with careful attention to dose, route, and hemodynamic effects. Close multidisciplinary planning and coordinated postpartum care are particularly important for women with pulmonary hypertension, ventricular dysfunction, complex congenital heart disease, and Fontan circulation.

## Introduction and background

Cardiovascular disease (CVD) is an increasingly important contributor to maternal morbidity and mortality. Improvements in the survival of patients with congenital heart disease, advances in treatment of acquired CVD, and increasing maternal age have resulted in a growing population of women entering pregnancy with clinically significant cardiovascular conditions [1,2]. Pregnancy itself imposes substantial cardiovascular demands, including increased blood volume and cardiac output, reduced systemic vascular resistance, and major hemodynamic changes during labor, delivery, and the postpartum period. These physiological adaptations may be poorly tolerated in patients with fixed cardiac output, ventricular dysfunction, pulmonary vascular disease, significant valvular obstruction, systemic or pulmonary venous abnormalities, or limited ability to augment cardiac output [1,2].

The anesthesiologist plays an important role throughout the peripartum period. Anesthetic decisions influence systemic vascular resistance, venous return, myocardial contractility, pulmonary vascular resistance, cardiac rhythm, ventilation, and oxygenation. These effects may have markedly different consequences depending on the underlying cardiac lesion. A decrease in systemic vascular resistance that is well tolerated in one patient may produce profound hypotension in a patient with severe aortic stenosis, whereas an increase in pulmonary vascular resistance that is modest in an otherwise healthy patient may precipitate right ventricular failure in a patient with pulmonary hypertension or Fontan circulation [2].

Contemporary management therefore emphasizes individualized risk assessment and multidisciplinary care. The 2025 European Society of Cardiology (ESC) guideline introduced the modified World Health Organization (mWHO) 2.0 classification, refined the role of the Pregnancy Heart Team, and incorporated additional clinical information into cardiovascular risk assessment [1]. The Cardiac Disease in Pregnancy (CARPREG) II model provides complementary quantitative risk estimation using general, lesion-specific, and care-related predictors of maternal cardiac complications [3]. Neither approach replaces individualized clinical assessment.

Despite existing guidelines, translating lesion-specific physiology into practical anesthetic decisions remains challenging. Questions concerning the choice and timing of neuraxial anesthesia, the role of invasive monitoring, the mode of delivery, management of anticoagulation, uterotonic selection, and postpartum surveillance often require interpretation beyond a single guideline statement. This review therefore focuses on practical anesthetic decision-making across major cardiovascular lesions encountered during pregnancy.

## Review

Review methodology

This article is a narrative clinical review rather than a systematic review or meta-analysis. The objective was to synthesize contemporary cardiovascular, obstetric, and anesthetic literature into a practical lesion-based framework rather than to estimate pooled treatment effects or perform formal comparative effectiveness analyses.

For this revision, targeted literature searches were updated through September 2026 using PubMed/MEDLINE, together with examination of major professional society guidelines, scientific statements, and relevant contemporary reviews and cohort studies. Searches focused on combinations of terms related to pregnancy, CVD, obstetric anesthesia, neuraxial anesthesia, cesarean delivery, pulmonary hypertension, valvular heart disease, aortic disease, congenital heart disease, Fontan circulation, cardiomyopathy, myocardial infarction, anticoagulation, uterotonics, and postpartum cardiovascular care. Particular attention was given to publications from recent years and to major guideline documents, while older studies were retained when they provided foundational lesion-specific anesthetic evidence that remains clinically relevant.

Sources were selected based on clinical relevance, methodological quality, recency, the authority of the issuing organization, and applicability to peripartum anesthetic management. Contemporary guidelines and scientific statements were prioritized for general recommendations, while lesion-specific cohort studies, case series, and anesthetic reports were used when direct evidence was available. Historical studies were retained when they provided important observations not adequately replaced by newer evidence. No formal study-level risk-of-bias assessment was performed because this was a narrative review.

The review does not follow PRISMA 2020 because it is not intended to represent a systematic review. The limitations inherent to a narrative approach, including potential selection bias and dependence on available observational evidence, should therefore be considered when interpreting the recommendations.

Cardiovascular risk assessment before delivery

Risk assessment should begin before delivery and, when possible, before conception. The contemporary approach combines lesion-specific clinical assessment with structured risk classification. Important features include ventricular function, functional status, symptoms, oxygen saturation, pulmonary pressures, arrhythmias, prior cardiac events, residual lesions, prosthetic valves, aortic dimensions, exercise capacity, anticoagulation, previous cardiac surgery, and the anticipated physiological response to labor and postpartum autotransfusion [1-3].

The 2025 ESC guideline introduced mWHO 2.0, expanding and refining the previous classification and incorporating a broader range of cardiovascular conditions [1]. Rather than functioning as a binary assessment of whether pregnancy should or should not occur, the classification is intended to support counseling, determine the level of specialist involvement, and guide the location and intensity of care. Women classified as mWHO 2.0 II-III or higher generally require involvement of a Pregnancy Heart Team, with the intensity of specialist care increasing with cardiovascular risk [1].

The mWHO classification should not be interpreted in isolation. A patient with the same anatomical diagnosis may have substantially different risk depending on ventricular function, symptoms, pulmonary pressures, arrhythmia burden, previous complications, and treatment. The 2025 ESC guideline emphasizes individualized counseling and shared decision-making, including for women in the highest risk categories [1].

The CARPREG II model provides complementary risk estimation [3]. Its predictors include prior cardiovascular events or arrhythmia, poor functional class or cyanosis, high-risk left-sided valve disease or left ventricular outflow tract obstruction, systemic ventricular dysfunction, absence of previous cardiac intervention when intervention would be expected to reduce risk, mechanical valve, high-risk aortopathy, pulmonary hypertension, coronary artery disease, and late pregnancy assessment [3]. CARPREG II therefore complements anatomical classification by incorporating clinical characteristics and care-related factors. Neither mWHO 2.0 nor CARPREG II should replace individualized clinical judgment.

For anesthetic planning, risk assessment should additionally answer practical questions: Can the patient tolerate the reduction in systemic vascular resistance associated with neuraxial anesthesia? Can she tolerate increased venous return after delivery? Is right ventricular function adequate to tolerate transient increases in pulmonary vascular resistance? Is anticoagulation compatible with neuraxial techniques? What degree of monitoring is required? Where should delivery occur, and what resources are immediately available if cardiovascular deterioration occurs?

Multidisciplinary planning and delivery pathway

High-risk cardiovascular pregnancy is best managed through coordinated multidisciplinary care, ideally beginning before delivery and continuing through the postpartum period. Contemporary guidelines emphasize the role of a Pregnancy Heart Team, with the composition and intensity of specialist involvement adapted to the severity and type of CVD [1,2,4]. The objective is not simply to assemble multiple specialists but to establish a coordinated plan that anticipates cardiovascular deterioration, obstetric complications, anesthetic requirements, and neonatal needs.

The core team generally includes cardiology, maternal-fetal medicine, obstetrics, obstetric anesthesiology, and specialized labor and delivery nursing. Additional services, including neonatology, critical care, hematology, cardiothoracic surgery, and transfusion medicine, should be involved when clinically indicated. Experience from specialized centers has demonstrated that even severe maternal heart disease does not invariably preclude favorable pregnancy outcomes when patients are appropriately assessed and managed in experienced multidisciplinary settings [5,6]. Their principal responsibilities are summarized in Table 1.

**Table 1:** Multidisciplinary pregnancy heart team: core roles and additional services according to clinical need Source: The table is based on contemporary guideline recommendations and multidisciplinary cardio-obstetric literature [1,2,4,6]

Team member/service	Principal role
Cardiology	Cardiovascular diagnosis, risk assessment, optimization, and longitudinal management
Maternal-fetal medicine	Maternal-fetal risk assessment, fetal surveillance, and obstetric planning
Obstetric anesthesiology	Preanesthetic assessment, labor analgesia, delivery anesthesia, hemodynamic planning, and perioperative care
Obstetrics and labor, and delivery nursing	Intrapartum monitoring, delivery coordination, and implementation of the delivery plan
Neonatology	Anticipation and management of neonatal complications when fetal or maternal risk is substantial
Critical care	Management of maternal hemodynamic or respiratory deterioration and postoperative critical care
Hematology	Anticoagulation, thrombosis, and bleeding management when relevant
Cardiothoracic surgery	Consultation for selected structural, aortic, or surgical emergencies
Transfusion medicine/blood bank	Blood-product planning and major hemorrhage support when indicated

The delivery plan should be individualized to the patient's cardiovascular lesion, current clinical status, anticipated physiological changes, and available institutional resources. Important elements include the intended mode and timing of delivery, location of delivery, anesthetic technique, vascular access, monitoring, anticoagulation management, availability of blood products, uterotonic strategy, and planned postpartum level of care. The team should also establish an escalation strategy for clinically important deterioration, including worsening ventricular function, hypoxemia, pulmonary hypertensive crisis, significant arrhythmia, severe hypotension, or major hemorrhage.

Anesthetic planning should begin before labor whenever possible. The anesthesiologist should review recent cardiac imaging, ventricular function, pulmonary pressures, functional status, rhythm, oxygenation, anticoagulation, previous cardiac procedures, and the patient's anticipated hemodynamic vulnerabilities. The principal cardiovascular goals should then be translated into a practical plan for labor analgesia, cesarean anesthesia if required, monitoring, vasoactive support, fluid management, and postpartum surveillance.

This process can be summarized as a practical peripartum pathway (Figure 1). The pathway is intended as a framework rather than a rigid algorithm; individual decisions should be modified according to the underlying lesion, severity of disease, maternal and fetal condition, urgency of delivery, and local expertise and resources.

**Figure 1:**
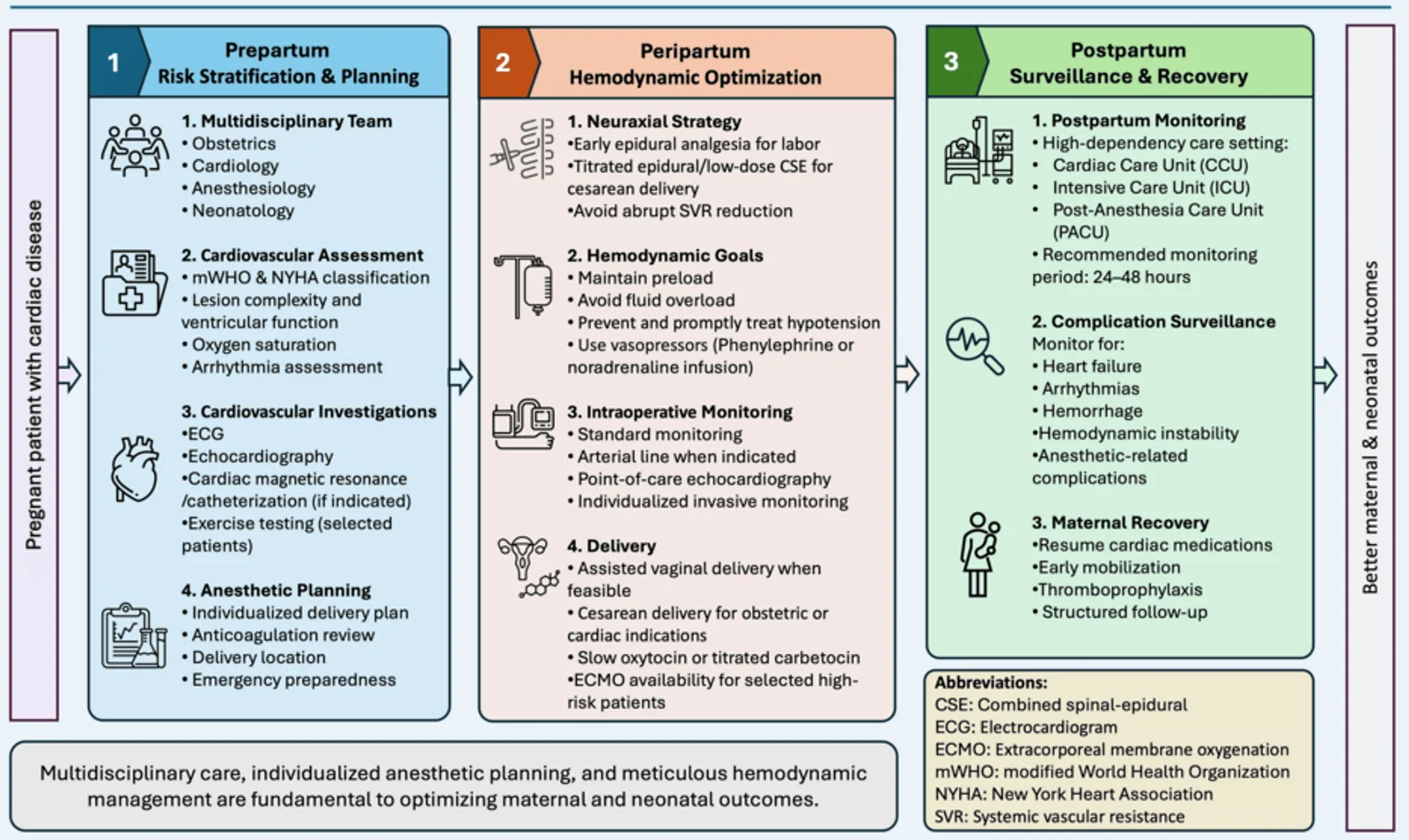
Practical multidisciplinary peripartum management pathway for pregnant patients with cardiovascular disease ECG: electrocardiogram; CSE: combined spinal-epidural; ECMO: extracorporeal membrane oxygenation; mWHO: modified World Health Organization; NYHA: New York Heart Association; SVR: systemic vascular resistance; CCU: cardiac care unit; ICU: intensive care unit; PACU: postanesthesia care unit Image credit: The image was created by the authors using Microsoft PowerPoint (Microsoft Corporation, Redmond, WA)

General hemodynamic principles of obstetric anesthesia

The fundamental principle of cardiovascular obstetric anesthesia is to preserve the physiological variables most important for the specific lesion. These generally include adequate preload, appropriate systemic vascular resistance, effective ventricular contractility, maintenance of sinus rhythm when atrial contribution is important, preservation of oxygenation and ventilation, and avoidance of abrupt changes in pulmonary vascular resistance [1,2,5,6].

Labor itself can produce substantial cardiovascular stress. Pain and anxiety increase sympathetic activity and may increase heart rate, blood pressure, and oxygen consumption. Uterine contractions intermittently reduce venous return, while autotransfusion following uterine contraction and placental separation increases venous return after delivery. Effective neuraxial labor analgesia may reduce catecholamine-mediated hemodynamic stress, but excessive sympathetic blockade can cause hypotension in patients with limited cardiovascular reserve [2,5].

Incremental neuraxial techniques are therefore often useful when cardiovascular physiology permits. Epidural analgesia allows gradual titration and can be extended for cesarean delivery when an established catheter is functioning adequately. Combined spinal-epidural techniques may provide rapid onset with the possibility of incremental epidural dosing, although the intrathecal component should be tailored to the patient's cardiovascular reserve. Single-shot spinal anesthesia may be appropriate for selected patients but can produce a relatively rapid decrease in systemic vascular resistance and therefore requires careful consideration in patients with fixed cardiac output or severe ventricular dysfunction [2,5].

General anesthesia remains an important component of the anesthetic armamentarium. It may be appropriate when neuraxial anesthesia is contraindicated, when anticoagulation prevents safe neuraxial anesthesia, when neuraxial anesthesia is inadequate, when rapid delivery is required, or when airway and ventilatory control are clinically necessary. The decision should be based on the patient's physiology and clinical circumstances rather than on a universal preference for either neuraxial or general anesthesia [2].

Positive-pressure ventilation can reduce venous return and alter pulmonary vascular and right ventricular loading conditions. These effects may be particularly important in patients with severe pulmonary hypertension, right ventricular dysfunction, or Fontan circulation. If general anesthesia is required, induction and ventilation should therefore be planned to minimize abrupt hemodynamic changes [2,5].

Monitoring should be proportional to cardiovascular risk. Standard maternal monitoring is appropriate for many patients with mild disease. Arterial pressure monitoring may be useful when rapid blood pressure changes are anticipated, vasoactive drugs are expected, or beat-to-beat hemodynamic information is clinically important. Central venous access is not routinely required, and central venous pressure should not be interpreted as a stand-alone measure of preload. Transesophageal echocardiography is similarly not routine but may be useful when transthoracic imaging is inadequate and real-time assessment of ventricular function, filling, or structural pathology would change management.

Uterotonic therapy and postpartum hemodynamics

Uterotonic therapy is an important component of obstetric anesthesia in patients with CVD because uterine contraction and management of postpartum blood loss occur during a period of substantial hemodynamic transition. Uterotonic agents differ in their effects on systemic vascular resistance, heart rate, myocardial contractility, and pulmonary vascular tone, and the clinical significance of these effects depends on the underlying cardiovascular lesion and its hemodynamic reserve [7,8] (Table 2).

**Table 2:** Uterotonic agents: cardiovascular considerations in patients with cardiac disease Source: This is an author-generated clinical summary based on contemporary literature and consensus recommendations [7,8]

Uterotonic agent	Cardiovascular considerations	Clinical considerations in patients with cardiac disease
Oxytocin	Rapid intravenous administration may cause vasodilation, decreased systemic vascular resistance, hypotension, and tachycardia	Commonly used as a first-line uterotonic. Controlled administration and careful titration may be preferable when abrupt hemodynamic changes are poorly tolerated
Carbetocin	Long-acting oxytocin analog with prolonged uterotonic activity. Hemodynamic effects remain clinically relevant and may depend on dose and rate of administration	May be considered in selected patients when prolonged uterine contraction is desirable. Evidence specifically in significant cardiac disease remains limited; administration should be individualized with attention to hemodynamic response
Methylergonovine/ergometrine	Produces vasoconstriction and may substantially increase systemic and pulmonary vascular resistance	Generally avoided or used only with specialist consideration when increased vascular resistance may be poorly tolerated, including in pulmonary hypertension, severe hypertension, significant obstructive lesions, or selected forms of ventricular dysfunction
Misoprostol	Generally causes less abrupt systemic hemodynamic change than vasoconstrictive uterotonics; may cause fever, shivering, nausea, and tachycardia	May be considered when other uterotonics are contraindicated or insufficient. Its role in severe cardiovascular disease is less well established
Carboprost	Can cause bronchospasm and may adversely affect pulmonary and cardiopulmonary physiology	Use cautiously or avoid when increases in pulmonary vascular load or bronchospasm would be poorly tolerated, particularly in patients with significant pulmonary hypertension or severe right ventricular dysfunction

Oxytocin remains a commonly used first-line uterotonic, but rapid intravenous administration can produce abrupt vasodilation, hypotension, and tachycardia. In patients who may tolerate these changes poorly, controlled administration of a carefully titrated dose may reduce abrupt hemodynamic effects while maintaining adequate uterine tone. Carbetocin has a longer duration of uterotonic activity and may reduce the need for additional uterotonics in selected settings, although evidence specifically addressing patients with significant CVD remains more limited than that for conventional oxytocin-based strategies [7,8].

Methylergonovine and related ergot alkaloids produce potent vasoconstriction and may increase systemic and pulmonary vascular resistance. They are therefore generally avoided, or used only after careful multidisciplinary consideration, when increases in vascular resistance could be poorly tolerated, including in patients with pulmonary hypertension, severe hypertension, significant obstructive cardiac disease, or selected forms of ventricular dysfunction. Carboprost may cause bronchospasm and may adversely affect pulmonary and cardiopulmonary physiology; consequently, its use warrants particular caution when increases in pulmonary vascular load or bronchospasm could compromise cardiovascular or respiratory function [7,8].

Misoprostol generally produces less abrupt systemic hemodynamic change than vasoconstrictive uterotonics and may be useful when other agents are unsuitable or insufficient. However, it can cause fever, shivering, nausea, and tachycardia, and its role in patients with severe CVD remains less well characterized [7,8].

Blood loss should be recognized and treated promptly because anemia, reduced venous return, and hypovolemia may be particularly poorly tolerated in patients with limited cardiovascular reserve. Conversely, indiscriminate volume replacement may also be harmful in patients susceptible to ventricular failure or pulmonary edema. Management of postpartum hemorrhage should therefore combine timely hemorrhage control with lesion-specific assessment of preload, systemic vascular resistance, ventricular function, and pulmonary vascular load. Decisions regarding escalation of uterotonic therapy, transfusion, vasopressor or inotropic support, and invasive monitoring should be individualized according to the severity and mechanism of cardiovascular compromise.

Anticoagulation and neuraxial anesthesia

Anticoagulation is common in pregnancy complicated by mechanical valves, atrial arrhythmias, Fontan circulation, venous thromboembolism, or other thrombotic risk. The decision to perform neuraxial anesthesia must therefore incorporate both the thrombotic risk of interruption and the bleeding risk associated with neuraxial procedures.

Timing of neuraxial placement and catheter removal should follow current evidence-based antithrombotic guidance rather than older lesion-specific case reports. The 2025 fifth edition of the American Society of Regional Anesthesia and Pain Medicine guidelines provides contemporary recommendations for neuraxial procedures in patients receiving anticoagulant and antiplatelet therapy [9]. Management should be coordinated with cardiology and hematology when interruption of anticoagulation carries substantial thrombotic risk.

The presence of a cardiovascular lesion does not itself contraindicate neuraxial anesthesia. Conversely, the potential cardiovascular benefits of neuraxial anesthesia do not justify violating established anticoagulation safety intervals.

Mitral stenosis

Mitral stenosis produces a fixed limitation to left ventricular filling and therefore depends on adequate diastolic filling time and controlled heart rate. Pregnancy increases plasma volume and cardiac output while reducing systemic vascular resistance. These changes can increase transmitral flow and left atrial pressure, potentially precipitating pulmonary edema in clinically significant disease [1,10].

The principal anesthetic objectives are to maintain adequate but not excessive preload, avoid tachycardia, preserve sinus rhythm when possible, and avoid abrupt increases in pulmonary vascular resistance. Hypoxemia, hypercarbia, acidosis, pain, and excessive sympathetic stimulation should be avoided.

Labor analgesia may be advantageous because it reduces pain-related tachycardia and catecholamine release. Neuraxial analgesia should be titrated carefully to avoid excessive hypotension. In severe disease, early placement of a functioning epidural catheter may provide greater flexibility should cesarean delivery become necessary.

For cesarean delivery, a carefully titrated neuraxial technique can be appropriate in selected patients. The choice between epidural, combined spinal-epidural, or another neuraxial approach should depend on disease severity and hemodynamic reserve. Severe pulmonary edema, marked pulmonary hypertension, significant right ventricular dysfunction, or clinical instability may require more intensive monitoring and individualized anesthetic planning.

Postpartum autotransfusion can increase preload abruptly. Patients with significant mitral stenosis may therefore remain at risk for pulmonary edema after delivery and should be observed according to clinical severity rather than automatically discharged from higher-acuity monitoring.

Aortic stenosis

Aortic stenosis creates a relatively fixed obstruction to left ventricular outflow. Cardiac output may depend on adequate preload, sinus rhythm, and maintenance of systemic vascular resistance. Excessive vasodilation can reduce coronary perfusion and systemic pressure, whereas tachycardia reduces diastolic filling and coronary perfusion [1,11].

The classic anesthetic objectives are therefore maintenance of adequate preload, sinus rhythm, controlled heart rate, and preservation of systemic vascular resistance. Sudden decreases in systemic vascular resistance should be avoided.

The peripartum anesthetic experience reported by Ioscovich et al. remains an important lesion-specific anesthesia reference [12]. Their series illustrates that anesthetic management should be individualized according to stenosis severity, symptoms, ventricular function, and mode of delivery rather than determined solely by the diagnosis of aortic stenosis [12].

In symptomatic or severe disease, invasive arterial pressure monitoring may be appropriate. A slowly titrated epidural or carefully selected combined spinal-epidural approach may be used in experienced hands when the patient is stable and hemodynamic changes can be controlled. General anesthesia may be required when neuraxial anesthesia is contraindicated or when urgent delivery or other clinical circumstances dictate.

Contemporary management should be informed by current pregnancy and valve guidelines rather than historical concerns that categorically prohibited neuraxial anesthesia in all severe aortic stenosis [1,11-13].

Aortic disease, coarctation, and Takayasu arteritis

Aortic disease encompasses a heterogeneous group of conditions, including aortic dilation associated with genetic syndromes, bicuspid aortic valve-associated aortopathy, previous aortic surgery, coarctation, and inflammatory aortitis. The major concern during pregnancy is the interaction between increased cardiac output, changes in vascular resistance, and mechanical stress on the aortic wall [1].

Patients with significant aortic disease require individualized assessment of aortic dimensions, lesion location, ventricular function, blood pressure, previous surgery, and the presence of residual obstruction or dissection. Avoidance of severe hypertension and large fluctuations in arterial pressure is generally desirable.

Coarctation requires attention to both proximal hypertension and distal perfusion. Blood pressure should be interpreted in the context of the anatomical lesion, and arterial monitoring may be considered when significant disease or major hemodynamic instability is anticipated [14].

Takayasu arteritis presents additional challenges because of large-vessel stenosis, blood pressure measurement discrepancies, vascular fragility, and possible coronary, renal, or cerebrovascular involvement. Ioscovich et al. described peripartum anesthetic management in a series of women with Takayasu arteritis and highlighted the importance of individualized hemodynamic management [15]. That report remains useful for lesion-specific anesthetic considerations, but contemporary recommendations regarding neuraxial anesthesia in anticoagulated patients should be based on current regional anesthesia guidelines [9].

Connective-tissue disorders such as Ehlers-Danlos syndrome may coexist with cardiovascular manifestations and create additional anesthetic concerns related to tissue fragility, bleeding, vascular complications, positioning, and airway or neuraxial considerations. A recent case report from our group illustrates the practical anesthetic considerations in a pregnant patient with Ehlers-Danlos syndrome undergoing elective cesarean delivery [16]. This case is best interpreted as lesion-specific clinical experience rather than evidence for general cardiovascular anesthetic management.

Pulmonary hypertension

Pulmonary hypertension represents one of the most challenging cardiovascular conditions in pregnancy. Increased pulmonary vascular resistance places a chronic pressure load on the right ventricle, while pregnancy and the postpartum period may further challenge right ventricular function. Hypoxemia, hypercarbia, acidosis, pain, sympathetic activation, and mechanical ventilation can all contribute to increased pulmonary vascular resistance [1,17-20].

Recent literature suggests that outcomes have improved compared with historical reports, particularly with modern multidisciplinary care and targeted pulmonary vasodilator therapy, but pulmonary hypertension remains associated with substantial maternal and fetal morbidity [17-20]. A contemporary nationwide analysis demonstrated that pulmonary hypertension remains associated with significant maternal and fetal complications despite advances in management [18]. A 2024 systematic review further demonstrated variation in pregnancy outcomes according to pulmonary hypertension severity and classification [17].

Anesthetic objectives include maintaining right ventricular perfusion and function, avoiding increases in pulmonary vascular resistance, preserving systemic blood pressure, maintaining adequate oxygenation and ventilation, and preventing sudden changes in preload.

Labor analgesia may reduce pain-associated increases in sympathetic activity and pulmonary vascular resistance. A carefully titrated epidural technique may therefore be considered in selected stable patients. However, the choice of neuraxial technique should consider right ventricular function, oxygenation, anticoagulation, baseline blood pressure, and delivery urgency. Neuraxial anesthesia should not be regarded as universally superior in every patient with pulmonary hypertension.

General anesthesia may be required when neuraxial anesthesia is contraindicated, when urgent delivery is necessary, or when airway and ventilatory control are clinically indicated. If general anesthesia is used, induction should be carefully planned to minimize systemic hypotension and increases in pulmonary vascular resistance. Mechanical ventilation should use the lowest effective mean airway pressures compatible with adequate gas exchange.

The mode of delivery should be individualized. Severe pulmonary hypertension may prompt consideration of planned cesarean delivery in selected patients, but there is insufficient basis for a universal rule that cesarean delivery is required for all patients with pulmonary hypertension [1,17,19,20]. Delivery planning should incorporate pulmonary pressures, right ventricular function, functional status, oxygenation, anticoagulation, obstetric factors, and the expertise of the treating center.

Postpartum care is particularly important because abrupt changes in venous return and vascular resistance can precipitate right ventricular failure. High-risk patients may require monitored or intensive care after delivery, with the duration determined by clinical severity and trajectory rather than a fixed universal observation period.

Pregnancy-associated myocardial infarction and coronary artery disease

Pregnancy-associated myocardial infarction is uncommon but potentially catastrophic. Mechanisms include spontaneous coronary artery dissection, atherosclerotic plaque rupture, coronary thrombosis, coronary vasospasm, and other pregnancy-associated conditions [21]. Management requires coordination among cardiology, obstetrics, anesthesia, and interventional cardiology.

The anesthetic approach depends on the timing of the infarction, ventricular function, ongoing ischemia, antiplatelet or anticoagulant therapy, coronary anatomy, and urgency of delivery. Hemodynamic stability and myocardial oxygen balance should be preserved. Tachycardia, significant hypotension, severe hypertension, hypoxemia, and excessive sympathetic stimulation should be avoided.

Neuraxial anesthesia may be appropriate in a stable patient when coagulation and antiplatelet therapy permit safe neuraxial procedures. When anticoagulation or dual antiplatelet therapy makes neuraxial anesthesia inappropriate, general anesthesia may be required. The risks of neuraxial bleeding must be balanced against the cardiovascular consequences of general anesthesia and positive-pressure ventilation [9,21].

Older literature contains recommendations concerning delaying delivery following myocardial infarction, but these recommendations should not be applied rigidly to contemporary patients. Delivery timing should be determined by maternal and fetal status, myocardial recovery, ongoing ischemia, and obstetric indications in collaboration with the multidisciplinary team.

Peripartum cardiomyopathy

Peripartum cardiomyopathy (PPCM) is characterized by left ventricular systolic dysfunction occurring toward the end of pregnancy or in the months following delivery. Clinical severity ranges from mild ventricular impairment to severe heart failure and cardiogenic shock. Contemporary risk assessment places severe left ventricular dysfunction and significant residual PPCM among the highest risk cardiovascular states in pregnancy [1,22].

Anesthetic management depends primarily on ventricular function and the degree of heart failure. The principal goals are avoidance of myocardial depression, excessive afterload, uncontrolled tachycardia, and abrupt volume shifts. Neuraxial techniques may be useful in stable patients because effective analgesia reduces sympathetic stress and avoids airway instrumentation, but sympathetic blockade should be titrated according to ventricular reserve.

Patients with severe ventricular dysfunction may require invasive arterial monitoring, vasopressor or inotropic support, and access to advanced heart-failure therapies. General anesthesia may be necessary in unstable patients or when urgent delivery is required. Postpartum surveillance is particularly important because the postpartum period can expose the ventricle to abrupt changes in preload and afterload.

Congenital heart disease

PPCM is characterized by left ventricular systolic dysfunction occurring toward the end of pregnancy or in the months following delivery. Clinical severity ranges from mild ventricular impairment to severe heart failure and cardiogenic shock. Contemporary risk assessment places severe left ventricular dysfunction and significant residual PPCM among the highest risk cardiovascular states in pregnancy [1,22,23].

Anesthetic management depends primarily on ventricular function and the degree of heart failure. The principal goals are avoidance of myocardial depression, excessive afterload, uncontrolled tachycardia, and abrupt volume shifts. Neuraxial techniques may be useful in stable patients because effective analgesia reduces sympathetic stress and avoids airway instrumentation, but sympathetic blockade should be titrated according to ventricular reserve.

Patients with severe ventricular dysfunction may require invasive arterial monitoring, vasopressor or inotropic support, and access to advanced heart-failure therapies. General anesthesia may be necessary in unstable patients or when urgent delivery is required. Postpartum surveillance is particularly important because the postpartum period can expose the ventricle to abrupt changes in preload and afterload.

Fontan circulation

Fontan circulation represents a distinctive cardiovascular physiology in which systemic venous blood reaches the pulmonary circulation without a subpulmonary ventricle. Pulmonary blood flow therefore depends on low pulmonary vascular resistance, adequate venous pressure, and effective respiratory mechanics. Pregnancy increases blood volume and cardiac output and may challenge a circulation with limited capacity to increase flow [24-26].

Pregnancy in women with Fontan circulation is associated with increased risks of arrhythmia, heart failure, thromboembolism, and adverse fetal outcomes. A 2025 systematic review and meta-analysis including 400 women and 1,260 pregnancies reported pooled maternal arrhythmia and heart failure rates of approximately 6.6% and 3.7%, respectively, with preterm delivery occurring in more than half of pregnancies [25]. A contemporary cohort study similarly demonstrated substantial fetal and maternal morbidity and emphasized the importance of specialist cardio-obstetric care [26].

The anesthetic goals are maintenance of venous return, preservation of low pulmonary vascular resistance, avoidance of excessive increases in intrathoracic pressure, maintenance of sinus rhythm, and prevention of systemic hypotension. Hypovolemia can reduce pulmonary blood flow and cardiac output, while excessive fluid administration may worsen ventricular filling pressures and congestion. Fluid management should therefore be guided by clinical assessment and, when available, echocardiographic information rather than by a fixed volume strategy.

Neuraxial analgesia can be advantageous because it provides effective labor analgesia without airway instrumentation and may reduce sympathetic activation. However, excessive vasodilation can reduce venous return and systemic pressure. Incremental epidural dosing or carefully titrated combined spinal-epidural techniques may therefore be considered in experienced centers.

An early report from Shaare Zedek Medical Center described emergency cesarean delivery using an indwelling epidural catheter in a woman with Fontan circulation and remains a useful illustration of the value of a functioning neuraxial catheter during an unplanned operative delivery [27]. More recent evidence has expanded the experience with neuraxial techniques. A 2015 report also described epidural anesthesia for cesarean delivery in a Fontan patient [28].

Our contemporary single-center study of 32 pregnancies in 12 women with Fontan circulation provides additional pregnancy-specific anesthetic data. Most cesarean deliveries were performed under neuraxial anesthesia, predominantly low-dose combined spinal-epidural anesthesia, with invasive arterial monitoring used frequently and phenylephrine used to support systemic pressure. No maternal deaths occurred in the study, although thrombotic complications occurred postpartum in two patients [29]. These data should be interpreted as single-center observational experience rather than evidence that one neuraxial technique is universally preferable.

Anticoagulation requires particular attention in Fontan patients because thrombosis is a recognized complication of the circulation and many patients receive antithrombotic therapy. Neuraxial procedures must therefore comply with contemporary anticoagulation guidance [9].

If cardiac arrest occurs, conventional advanced life support principles should be followed while rapidly addressing Fontan-specific reversible causes, including hypovolemia, arrhythmia, hypoxemia, increased pulmonary vascular resistance, and obstruction of Fontan pathways. Early multidisciplinary activation is important. Where available and appropriate, extracorporeal cardiopulmonary resuscitation may be considered when conventional resuscitation fails.

Practical peripartum framework

A practical approach to a pregnant patient with CVD begins with a predelivery assessment that identifies the cardiovascular lesion, current ventricular function, functional status, rhythm, oxygenation, pulmonary pressures, anticoagulation, previous cardiac procedures, and anticipated hemodynamic vulnerabilities. The anesthesiologist should then define the principal physiological goals and agree with the multidisciplinary team on the intended mode and timing of delivery.

During labor, analgesia should reduce pain and sympathetic activation while avoiding excessive sympathetic blockade. Early placement of neuraxial analgesia can be useful in patients in whom urgent operative delivery would be hazardous or in whom general anesthesia may be poorly tolerated, provided there is no contraindication.

For cesarean delivery, the choice of anesthetic technique should follow from the cardiovascular physiology rather than from the cardiac diagnosis alone. Neuraxial anesthesia is frequently feasible, but the technique and dosing strategy should be individualized. General anesthesia should remain readily available when neuraxial anesthesia is contraindicated, inadequate, too slow for the clinical situation, or otherwise inappropriate.

Hemorrhage management requires simultaneous attention to obstetric and cardiovascular physiology. Blood loss should be treated promptly, but indiscriminate fluid loading should be avoided in patients susceptible to pulmonary edema or ventricular failure. Uterotonic agents should be selected according to their cardiovascular effects and administered in a titrated manner when clinically appropriate [7,8].

Following delivery, the anesthetic team should anticipate the hemodynamic effects of autotransfusion and the rapid changes in systemic and pulmonary vascular resistance. Patients with severe pulmonary hypertension, significant ventricular dysfunction, severe valvular disease, complex congenital heart disease, or Fontan circulation may require monitored or intensive care. The intensity and duration of monitoring should be individualized according to cardiovascular risk and clinical trajectory.

Postpartum management

The postpartum period is an extension of cardiovascular risk rather than the end of peripartum management. Removal of placental circulation, uterine contraction, autotransfusion, changes in vascular tone, fluid shifts, pain, and hemorrhage can rapidly alter cardiovascular loading conditions [1,2].

Patients with left ventricular dysfunction are at risk of pulmonary edema and heart failure. Patients with pulmonary hypertension or Fontan circulation are vulnerable to changes in venous return and pulmonary vascular resistance. Patients with significant aortic disease may remain at risk from hypertension, while patients with arrhythmia-prone lesions may develop clinically significant tachyarrhythmias during the postpartum period.

There is no universal requirement for 24-48 hours of intensive monitoring in every patient with CVD. Instead, monitoring should reflect the lesion, ventricular function, symptoms, mode of delivery, blood loss, anesthetic course, vasoactive drug requirement, and immediate postpartum trajectory. Patients with severe pulmonary hypertension, significant ventricular dysfunction, recent cardiac events, or unstable physiology may appropriately require intensive or cardiac monitoring for at least the early postpartum period.

Postpartum care should also address anticoagulation resumption, heart-failure therapy, blood pressure management, thromboembolic risk, breastfeeding-compatible medication selection, and contraception. Longer term cardiovascular follow-up is important because adverse pregnancy outcomes may identify women at increased future cardiovascular risk [30]. The postpartum period therefore represents an opportunity not only to detect immediate complications but also to establish long-term cardiovascular prevention and specialist follow-up.

Considerations in resource-limited settings

The principles of cardiovascular obstetric anesthesia remain applicable when advanced monitoring or extracorporeal support is unavailable. In such settings, careful predelivery assessment, reliable intravenous access, continuous pulse oximetry and blood-pressure monitoring, early recognition of deterioration, judicious fluid administration, and immediate availability of vasoactive medications may be more important than the availability of highly specialized equipment.

For patients with severe CVD, transfer to a center capable of providing multidisciplinary cardiovascular, obstetric, neonatal, and critical care should be considered whenever feasible. When transfer is not possible, a clearly documented escalation plan should identify the most likely complications and available interventions.

Limitations

This review has several limitations. First, it is a narrative rather than systematic review and therefore may be affected by selection bias. Second, evidence for many lesion-specific anesthetic decisions consists primarily of retrospective cohorts, case series, and expert consensus rather than randomized clinical trials. Third, CVD encompasses heterogeneous lesions for which general recommendations cannot capture all clinically relevant differences. Fourth, contemporary guidelines increasingly emphasize individualized decision-making, making rigid anesthetic algorithms inappropriate for many high-risk patients. Finally, advances in cardiovascular and obstetric care mean that historical outcome data may not accurately represent outcomes in contemporary multidisciplinary centers.

## Conclusions

The anesthetic management of pregnant patients with CVD should be based on lesion-specific physiology, contemporary cardiovascular risk assessment, and coordinated multidisciplinary planning. The mWHO 2.0 classification and CARPREG II provide complementary frameworks for identifying patients who require specialist care, but neither replaces individualized clinical assessment.

Across cardiovascular lesions, the central anesthetic objective is preservation of the physiological variables on which cardiac output depends. These may include preload, systemic vascular resistance, sinus rhythm, ventricular contractility, oxygenation, and low pulmonary vascular resistance. Neuraxial anesthesia can provide important benefits for selected patients, particularly when gradual titration and effective labor analgesia are desirable, but its use must be individualized according to cardiovascular reserve and anticoagulation status. General anesthesia remains appropriate when neuraxial anesthesia is contraindicated or inadequate, rapid delivery is required, or maternal and surgical circumstances dictate.

Pulmonary hypertension, severe ventricular dysfunction, complex congenital heart disease, and Fontan circulation require particularly careful planning because relatively small changes in preload, afterload, ventilation, or pulmonary vascular resistance can produce major clinical consequences. Uterotonic therapy should similarly be individualized, with careful attention to the cardiovascular effects of each agent rather than reliance on universal avoidance or preference.

Finally, delivery should not be considered the endpoint of cardiovascular care. The postpartum period is characterized by major hemodynamic changes and remains a period of potential decompensation. An individualized postpartum monitoring and follow-up strategy, coordinated by the Pregnancy Heart Team, is therefore an integral component of safe anesthetic care.
